# Respiratory Infections Cause the Release of Extracellular Vesicles: Implications in Exacerbation of Asthma/COPD

**DOI:** 10.1371/journal.pone.0101087

**Published:** 2014-06-27

**Authors:** Suffwan Eltom, Nicole Dale, Kristof R. G. Raemdonck, Christopher S. Stevenson, Robert J. Snelgrove, Pradeep K. Sacitharan, Chiara Recchi, Silene Wavre-Shapton, Daniel F. McAuley, Cecilia O'Kane, Maria G. Belvisi, Mark A. Birrell

**Affiliations:** 1 Respiratory Pharmacology, Imperial College, London, United Kingdom; 2 Novartis, Horsham, United Kingdom; 3 Leukocyte Biology, Imperial College, London, United Kingdom; 4 Molecular Medicine, Imperial College, London, United Kingdom; 5 Institute of Ophthalmology, University College London, London, United Kingdom; 6 Centre for Infection and Immunity, Queen's University of Belfast, Belfast, United Kingdom; 7 MRC-Asthma UK Centre in Allergic Mechanisms of Asthma, Imperial College, London, United Kingdom; University of Hong Kong, Hong Kong

## Abstract

**Background:**

Infection-related exacerbations of respiratory diseases are a major health concern; thus understanding the mechanisms driving them is of paramount importance. Despite distinct inflammatory profiles and pathological differences, asthma and COPD share a common clinical facet: raised airway ATP levels. Furthermore, evidence is growing to suggest that infective agents can cause the release of extracellular vesicle (EVs) *in vitro* and in bodily fluids. ATP can evoke the P2X_7_/caspase 1 dependent release of IL-1β/IL-18 from EVs; these cytokines are associated with neutrophilia and are increased during exacerbations. Thus we hypothesized that respiratory infections causes the release of EVs in the airway and that the raised ATP levels, present in respiratory disease, triggers the release of IL-1β/IL-18, neutrophilia and subsequent disease exacerbations.

**Methods:**

To begin to test this hypothesis we utilised human cell-based assays, *ex vivo* murine BALF, *in vivo* pre-clinical models and human samples to test this hypothesis.

**Results:**

Data showed that in a murine model of COPD, known to have increased airway ATP levels, infective challenge causes exacerbated inflammation. Using cell-based systems, murine models and samples collected from challenged healthy subjects, we showed that infection can trigger the release of EVs. When exposed to ATP the EVs release IL-1β/IL-18 via a P2X_7_/caspase-dependent mechanism. Furthermore ATP challenge can cause a P2X_7_ dependent increase in LPS-driven neutrophilia.

**Conclusions:**

This preliminary data suggests a possible mechanism for how infections could exacerbate respiratory diseases and may highlight a possible signalling pathway for drug discovery efforts in this area.

## Introduction

Asthma and Chronic Obstructive Pulmonary Disease (COPD) are respiratory diseases with ever-increasing global prevalence [1]–[3] that represent a social and economic burden for industrialised and developing countries [4]. The World Health Organization currently states the number of patients suffering from asthma is 300 million and predicts this figure to rise to 400 million by 2025 [5], whereas there are 600 million COPD sufferers worldwide and the disease is predicted to be the third ranked leading cause of death by 2020 [5].

Exacerbations are common events in the lives of patients with asthma and COPD [6]–[8]. These episodes are often associated with infections by viruses or bacteria [9] and cause worsening of symptoms, which can be fatal. Often these heightened symptoms are far less responsive to normal treatments and are associated with increased health care costs and societal impact [10]. Increases in inflammatory status, particularly IL-1β and neutrophilia, in the airway are evident during exacerbations of both diseases [9], [11]–[16]. Furthermore, there is increasing evidence to suggest that the exacerbations accelerate the progressive decline in lung function [8], [11]. Therefore there is an urgent need to understand the mechanisms driving exacerbations and identify novel therapeutic interventions to target this cohort of patients.

Extracellular vesicles (EV) such as exosomes and microvesicles have been shown to be released from a diverse range of cell types in response to infective agents/pathogens and are believed to primarily function in immune surveillance and host defence (recently reviewed [17]–[19]. These vesicles contain proteins, lipids, mRNA and microRNA; they typically range from 30 nm to 1 µm in size and are found in many biological fluids. Recent cell-based studies have shown that ATP-stimulated EVs release IL-1β and IL-18 via the P2X_7_/caspase-1 axis [20]–[23] and it is known that these cytokines are involved in airway neutrophilia, activation of macrophages and the maintenance of a chronic inflammatory response [4]. Furthermore, it has been reported that ATP levels are increased in the airways of patients with asthma and COPD [24]–[27]. Indeed, despite distinct inflammatory and pathological patterns, raised ATP levels in asthma and COPD represents one common clinical attribute. Therefore our hypothesis is that exacerbations of asthma and COPD during respiratory infections are due to ATP (a known danger associated molecular pattern) activating the P2X_7_/caspase-1 axis within EVs resulting in the release of IL-1β and IL-18, and subsequently increasing neutrophilia and worsening of symptoms that may accelerate disease pathogenesis. Initial cell based data confirmed previous findings that a bacterial mimetic can cause the release of EVs [23] and indicated to us that we could use the ATP driven release of IL-1β as a marker of the presence of EVs in our biological samples. We then used Electron Microscopy (EM), Nanosight Technology and pharmacology to show that inhaled endotoxin causes the release of EVs in the airways of mice and man. Furthermore, parallel EV release in the airway could be triggered with live bacteria and a viral mimetic. Finally we showed that exogenous ATP can trigger a P2X_7_ receptor dependent exacerbated response to inhaled bacterial mimetic.

## Materials and Methods

### Demonstration that bacterial mimetic (LPS)-induced release of EVs can enhance IL-1β and neutrophil levels and change disease phenotype in model known to have increased levels of ATP

To begin to investigate our hypothesis we first determined if we could model the exacerbated inflammatory response to inhaled infective agent. Recently, its been shown that inhaled bacterial mimetic, LPS, causes greater inflammation if rats have been exposed to cigarette smoke [22]. We, and others, have shown that cigarette smoke causes an increase in airway ATP levels [21], [23], indeed it seems likely that the ATP, acting on P2X_7_ receptor, that is driving the inflammation after exposure to cigarette smoke [24], [25]. Thus it is conceivable that the exacerbated neutrophilia reported by Hardaker *et al.* [22] could be through the ATP-EV-IL-1β axis. As much of the recent work performed by our group and our collaborators is performed using mice, we first wanted to repeat the study reported by Hardaker *et al.* (2010) in mice.

All animal procedures were approved by the British Home Office, under the United Kingdom Animal (Scientific Procedures) Act 1986 (Project license 70/7212). Male adult C57bl/6 mice (18–25 gm) were purchased from Harlan UK Ltd and maintained in cages under controlled temperatures (19–23°C) and lighting (12-h light, 12-h dark cycle, lights on at 07:00 hrs). All mice had *ad libitum* access to water and chow (RM1 diet; Special Diet Services, Devon, UK).

The protocol for this study was designed specifically to examine the effect of the combination of the two stimuli. We focused not on the optimum time for smoke induced inflammation but when we knew the ATP levels were increased. Mice (*n* = 6 per treatment group) were exposed to either room air (control) or CS (3R4F cigarettes; Tobacco Health Research Institute, University of Kentucky, Lexington, KY, USA) using a negative pressure system as previously described [28], [29]. Mice were subjected to 2 periods of CS exposure (500 ml/minute) per day (4 hours apart) for 3 consecutive days. On the morning of the third day, the mice were challenged with aerosolised vehicle of endotoxin-free saline (Fresenius Kabi Ltd, Warrington, UK) or LPS (1 mg/ml; to mimic bacterial infection) in Perspex chambers for 30 minutes (as previously described, [28], [30]. Animals were culled and BALF and lung tissue samples were collected 24 hours after LPS treatment. IL-1β levels were measured in the BALF and neutrophil numbers determined in the BALF and lung tissue digest as described in Birrell *et al.* and Eltom *et al.* [28], [31].

BALF ATP levels were determined in samples from separate parallel smoke and LPS driven studies using an ATPlite luminescence assay.

### Demonstration of the concept that an infective insult can cause EV release

To confirm that it was appropriate to use ATP induced IL-1β as a biological marker of functional EV presence we used a cell based system [23]. THP-1 (Human acute monocytic leukemia cell line) monocytes were obtained from the European Collection of Cell Cultures (Wiltshire, UK), cultured in Roswell Park Memorial Institute medium (RPMI) 1640 medium plus GlutaMAX-I and supplemented with 10% foetal calf serum and 1% Penicillin/Streptomycin/Amphotericin B. Cells were maintained under sterile conditions in a humidified atmosphere of 37°C containing 95% air, 5% (v/v) CO_2_. Cells were cultured 10^6^ per T-75 culture flasks (Corning Inc, NY, USA). Trypan Blue exclusion was performed to determine cell viability. Cells were then incubated for one hour at 37°C in a humidified atmosphere (95% air; 5% (v/v) CO_2_) in order to settle. Flasks were treated with RPMI (vehicle) or LPS (0.1 µM, final concentration of *Escherichia coli* serotype 0111:B4 (Sigma-Aldrich Co)) and incubated overnight at 37°C (95% air; 5% (v/v) CO_2_). Eighteen hours after treatment, samples were collected and centrifuged at 900 g for 10 minutes to remove the cells. The supernatants were collected and split into two equal fractions. One fraction (EV-rich) was stored at −80°C without further processing. The other fraction was ultracentrifuged at 100,000×g for 2 hours (4°C) to remove the EVs. The supernatant (EV-deficient) was stored at −80°C for further experiments. Supernatant samples from both fractions were thawed at room temperature and 100 µl of each sample was pre-treated with vehicle (DMSO, final concentration 0.1%, V/V) or P2X_7_ antagonist (AZ 11645373; 10^−7^ M; AstraZeneca Pharmaceuticals PLC (AZ), UK – concentration established in Eltom *et al.* 2011). Samples were incubated for one hour (37°C; 95% air; 5% (v/v) CO_2_) and then subsequently treated with RPMI (vehicle) or Adenosine 5′-[*γ*-thio]triphosphate tetralithium salt (ATP*γS*; Sigma-Aldrich Co; 10^−3^ M – concentration established in Eltom *et al.* 2011). Thereafter, the samples were incubated for 4 hours (37°C; 95% air; 5% (v/v) CO_2_) prior to ELISA assessment for cytokines linked to the P2X_7_-inflammasome axis, IL-1β and IL-18, and control non-inflammasome markers such as TNFα and MMP-9. ELISAs used were purchased from R&D Systems Europe Ltd, Oxfordshire, UK.

### Determining if a bacterial mimetic can cause the release of EVs in the lung

Having demonstrated the concept that a bacterial mimetic insult can cause the release of EVs and IL-1β is useful biological marker of their presences, we then wanted to determine if a range of infective mimics/agents would cause the release of EVs in the airway. To do this we challenged mice with bacterial and viral mimetics, collected the lavage fluid and examined ATP-induced EV release of IL-1β (IL-18 was not measured in all experiments because of the limited sample obtained and the cost of the assays).

### Bacterial mimetic: LPS

Mice (*n = 6* per group) were challenged with the aerosolised vehicle or LPS as described above. Animals were sacrificed and BALF samples were obtained 6 hours after challenge.

To assess the presence of EV's in the BALF they were first centrifuged (900 g) to remove the white blood cells and debris. Samples were then:

### Visualisation of extracellular vesicles by electron microscopy

BALF was collected from a parallel set of mice described above, the white cells and debris were removed by centrifugation (3000 g) and then the EVs collected via ultracentrifugation (100,000 g). A drop of the resuspended EVs was placed on Formvar carbon-coated copper electron microscopy grids. Samples were subsequently fixed in 4% paraformaldehyde/1% gluteraldehyde in phosphate buffer, washed 10 times with ddH_2_O, stained with 1% methyl cellulose/2% uranyl acetate pH 4 on ice and visualised with a JEOL 1010 Transmission Electron Microscope (Welwyn Garden City, UK). Images were taken with a Gatan Orius SC100B charge-coupled device camera.

### Visualisation of extracellular vesicles by Nanosight Technology

The Nanosight (Amsebury, UK) system has recently been suggested to be the most appropriate way of assessing the size profile of EVs in biological fluids [32], [33]. It was used under the guidance of one of their trained technicians and made possible through a grant from The Rosetrees Trust, (A523).

### Quantitating extracellular vesicles by measuring ATP induced cytokine release

Samples were treated with RPMI (vehicle) or exogenous ATP*γS* (Sigma-Aldrich; 10^−3^ M, concentration established previously,[28]) and incubated for a further 4 hours (37°C; 95% air; 5% (v/v) CO_2_). Samples were then collected and stored for cytokine analysis by specific ELISA. Levels of IL-1β, IL-1α and IL-18 were determined.

### Assessing the role of P2X_7_ and caspase in the release of IL-1b

Samples were pre-treated with inhibitors (P2X_7_ antagonist A438079 (10^−6^ M); Abbot Laboratories and caspase-1 inhibitor VX-765 (10^−7^ M); Vertex Pharmaceuticals, Oxfordshire, UK) and incubated for 1 hour. For these experiments we used the Abbott compound because we have previously found that, unlike the AZ compound, it is effective antagonist of the murine P2X_7_ receptors [28]. Thereafter, RPMI (vehicle) or exogenous ATP*γS* (Sigma-Aldrich; 10^−3^ M) were added to the samples and incubated for a further 4 hours (37°C; 95% air; 5% (v/v) CO_2_). Samples were then collected and stored for cytokine analysis by ELISA. TNFa was measured as a negative control.

### LPS challenge in healthy subjects

To translate our murine findings we repeated some of the assessment in BALF samples collected from healthy human subjects challenged with vehicle (n = 5) or LPS (n = 10). Samples were generated as described in a study by Shyamsundar [34]. Shyamsundar *et al.* reported that “The study was approved by the local research ethics committee, and written informed consent was obtained from all subjects before enrolment in the study”. Briefly, LPS (Escherichia coli serotype O26:B6) was dissolved in endotoxin-free sterile 0.9% saline and inhaled via an automatic inhalation–synchronized dosimeter nebulizer (Spira). The total dose of inhaled LPS was 50 mg. Bronchoalveolar lavage (BAL) was performed 6 hours after vehicle of LPS challenge. Cells were removed via centrifugation (900 g) and the BALF supernatant processed as described above.

BALF ATP levels were determined in samples from separate parallel smoke and LPS driven studies using an ATPlite luminescence assay.

### Live bacterial challenge: *H. influenzae*

To show that live bacterial challenge causes the release of EVs, mice were infected with *Haemophilus influenzae* (H. *influenzae*). Briefly, Hib Eagan strain was a kind gift from P. Langford (Faculty of Medicine, St Mary's Hospital, Imperial College London). Bacteria were cultured at 37°C in 5% CO_2_ in Brain heart Infusion broth (OXOID) supplemented with 10 µg/ml of both Hemin (10ug/ml) and Nictinamide adenine dinucleotide (NAD) (Sigma-Aldrich, UK) or on BHI agar (OXOID) supplemented with 4% Levinthals when agar was <50°C. Levinthals was made by adding 50% horse blood (TCS Biosciences) to BHI broth and heating to 70°C for 45 minutes. On cooling to 50°C, 0.7 mg/ml NAD was added and the supernatant stored at –80°C. Bacteria were cultured to an OD600 of 0.3 (approximately 1×10^9^ CFU/ml) and stored at −80°C in 10% glycerol as single use aliquots.

Groups of mice (*n* = 4–5 per group) were infected i.n. with 1×10^7^ colony forming units of *H. influenzae* serotype b (strain Eagan) in sterile phosphate buffered saline (PBS). Terminal anaesthesia was induced at 6, 24 and 72 hours after challenge. Mice were culled and BALF samples were collected at 6, 24 and 48 hours. Presence of EVs was assessed as detailed above.

### Viral mimetic: Poly IC

To determine if viral challenge would cause EV we challenged mice with vehicle or the viral mimetic of Polyinosinic: polycytidylic acid (Poly I:C) as previously described [35]. Briefly, mice (*n* = 6 per group) were anesthetized with isoflurane (4% isoflurane in oxygen; Abbot Laboratories, Maidenhead, UK) and were intranasally (i.n.) administered with 50 µl (approximately 25 µl per nostril) of either saline (vehicle) or a 0.6 mg/ml solution of Poly I:C (Invitrogen Ltd, UK). Mice were culled and BALF samples were collected at 2, 6 and 24 hours. Presence of EVs was assessed as described above.

### Determining whether the ATP/P2X_7_ axis is central to the exacerbation response *in vivo*

Finally, experiments were performed to show that the ATP/P2X_7_ axis can exacerbate responses to inhaled bacterial mimetic *in vivo.* We were not able to use the smoke plus LPS exacerbation model systems because we, and others, have shown that ATP and P2X_7_ are central to the smoke only model, thus interpretation of any agents designed to modulate ATP/P2X_7_ would be difficult [27], [28]. Previously, we have shown that the inflammation in the basic LPS model is not modulated by a P2X_7_ inhibitor (22), thus it was ideal to use as a mechanistic model for testing the hypothesis that the P2X_7_ receptor was involved in the ATP-driven exacerbation. Briefly, the mice were challenged with vehicle or LPS, and four hours later intranasally dosed with saline (2 ml/kg) or ATPγs (0.001 mg/kg, Sigma, UK) whilst under light anaesthesia (4% isoflurane in oxygen). The mice received oral vehicle or P2X_7_ inhibitor, A438079 (1000 mg/kg), 30 minutes prior to the ATP challenge, 4 hours after the challenge and 1 hour prior to cull. Twenty Four hours after the LPS exposure the mice were culled and lavaged. Neutrophils numbers were measured in the BALF as described in Eltom et al. [28].

### Data analysis

All data are expressed as mean +/− standard error of mean (S.E.M) of *n* observations. Experiments were statistically analysed utilising the Students unpaired t-test for parametric data and the Mann-Whitney U-test for non-parametric data with independent groups compared with their specific controls or time-matched controls. For multiple comparison tests, statistical analysis was performed by applying One-way analysis of variance (ANOVA) followed by a Bonferroni's multiple comparison post-test for parametric data or a Kruskal-Wallis test incorporating a Dunn's multiple comparison post-test for non-parametric data. A *P* value of <0.05 was accepted as significant. Data analysis was performed using GraphPad Prism 5.0.

## Results

### LPS-induced release of EVs exacerbates ATP associated airway inflammation

**Figure 1:**
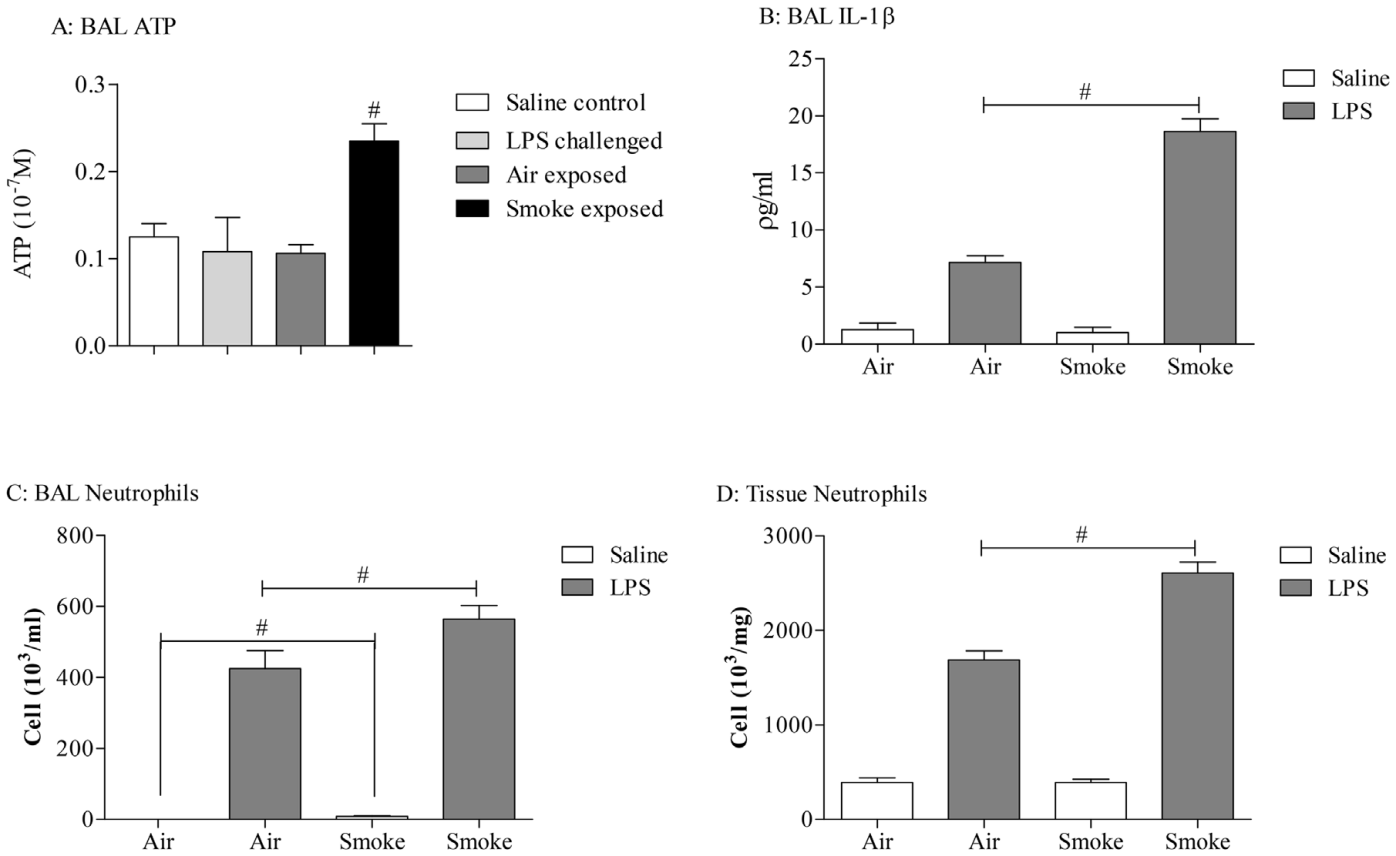
Demonstration that LPS-induced release of EVs can enhance IL-1β and neutrophil levels and change disease phenotype in model known to have increased levels of ATP. Mice (n = 8 per treatment group) were exposed to either room air (control) or CS (3R4F cigarettes) using a negative pressure system. Mice were subjected to 2 periods of CS exposure (500 ml/minute) per day (4 hours apart) for 3 consecutive days. On the morning of the third challenge day, the mice were exposed to aerosolised vehicle of endotoxin free saline or LPS (1 mg/ml) in Perspex chambers for 30 minutes. Animals were culled and BALF and lung tissue samples were collected 24 hours after LPS treatment. IL-1β levels were measured in the BALF and neutrophil numbers were determined in the BALF and lung tissue. In separate BALF samples collected from parallel smoke or LPS driven challenges ATP levels were measured (Panel A). Data shown as mean +/− S.E.M. (A: ATP #  = P = 0.0023, Mann-Whitney; B: IL-1β #  = P = 0.0009, Mann-Whitney; C: BALF neutrophil number, #  = P = 0.0431, Students T test; D: lung tissue neutrophil number; #  = P = 0.0006, Mann-Whitney).

LPS challenge caused an increase in BALF IL-1β levels, neutrophils and lung tissue neutrophils. Utilising mice that had been previously exposed to cigarette smoke, and thus had increased ATP levels in the BALF, the response to LPS was significantly exacerbated (Figure 1B–D). This suggests that the mouse is an appropriate species to model the exacerbation responses and thus can be used for further investigations into infection induced release of EVs.

BALF ATP levels were increased in samples from a separate parallel smoke driven study but not after LPS challenge (Figure 1A).

### Demonstration of the concept that an infective insult can cause EV release

**Figure 2:**
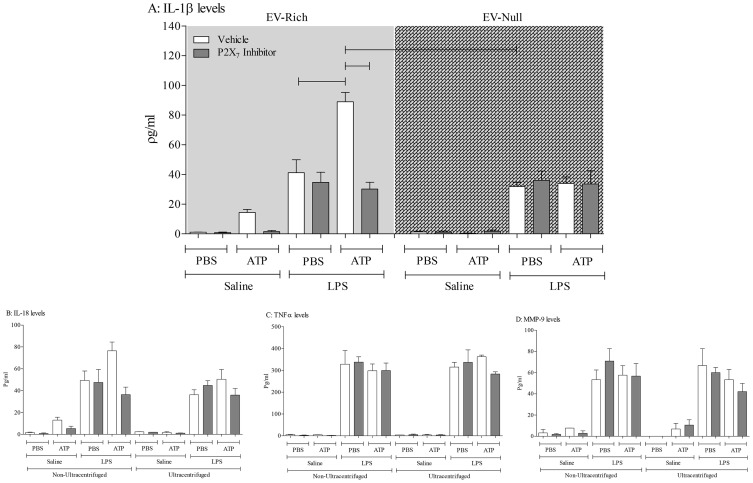
Demonstration of the concept that a bacterial mimetic insult can cause EV release. THP-1 cells were treated with RPMI (vehicle) or LPS (0.1 µM) and incubated overnight and samples were collected and centrifuged to remove the cells. The _supernatants_ were collected and split into two equal fractions: non-ultracentrifuged (EV-rich – left side) and ultracentrifuged (EV-_deficient – right side_). The samples were pre-treated with vehicle (DMSO, 0.1%, V/V) or P2X_7_ antagonist (AZ 11645373; 10^−7^ M). Samples were incubated for one hour and then treated with vehicle (PBS) or exogenous ATP*γS* (10^−3^ M). The samples were then incubated for a further 4 hours prior to ELISA assessment for cytokines (A: IL-1β, B: IL-18, C: TNFα, D: MMP-9). The data is shown as mean +/− S.E.M.

Before using the *in vivo* model systems, we initially wanted to use a cell based system to demonstrate the concept that an infective insult can cause the release of EVs and that subsequent challenge of these EVs with ATP will drive the P2X_7_-dependent release of IL-1β/IL-18. Treating cultured THP-1 cells with a bacterial mimetic, LPS, caused an increase in supernatant levels of IL-1β, IL-18, TNFα and MMP-9 (Figure 2). As expected the levels of these mediators were not altered by ultracentrifugation, nor were they altered when the P2X_7_ inhibitor was added to the cell-free supernatants (Figure 2). Interestingly, it would appear that cultured cells release some EVs under “basal” conditions. As can be seen in Figure 2, ATP triggers some P2X_7_-dependant IL-1b release. This could imply that cultured THP-1 cells release EVs constantly or that it is a consequence of culturing procedures.

Treatment of the cell-free supernatants with ATP increased the levels of IL-1β and IL-18 but not TNFα and MMP-9 in the EV-rich (non-ultracentrifuged) LPS challenged samples (Figure 2). This increase was attenuated when the P2X_7_ inhibitor, which suggests this receptor is central to the response. Significantly, when the samples were ultracentrifuged to remove the EVs, the ATP-induced increase in IL-1β/IL-18 was lost (Figure 2, right side of panels). Together this data shows that LPS can cause the release of EVs which in turn can release IL-1β/IL-18 after ATP stimulation in a P2X_7_ receptor dependent manner. Furthermore, importantly this data suggests that measuring ATP induced IL-1β in cell free medium is an appropriate surrogate marker of the presence of viable/functional EVs.

### Determining whether infective agents can cause the release of EVs in the lung

Having demonstrated the concept that an infective insult can cause the release of EVs in a cell culture system, we then wanted to determine if this occurred *in vivo*. To do this we challenged mice with LPS, collected the lavage fluid and performed EM, Nanosight assessment and examined ATP-induced IL-1β production. The inflammatory response after LPS challenge was typical of that previously described [28], [30].

**Figure 3:**
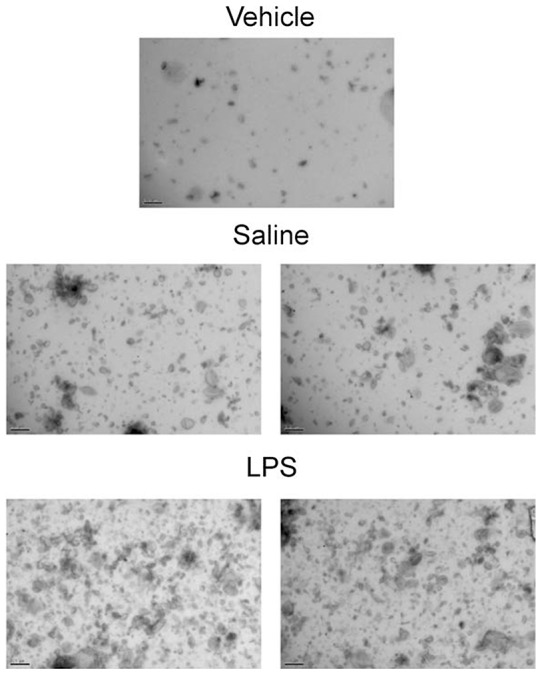
Determining if a bacterial mimetic (LPS) can cause the release of EVs in the lung – Electron Microscopy. Mice were challenged with the aerosolised vehicle of endotoxin-free saline or LPS (1 mg/ml) in Perspex chambers for 30 minutes. Animals were sacrificed and BALF obtained 6 hours after challenge. The samples were then centrifuged (900 g) to remove the white blood cells and debris. The presence of EVs was imaged using EM (top panel – vehicle, middle panels – vehicle challenge, bottom panels – LPS challenge).

**Figure 4:**
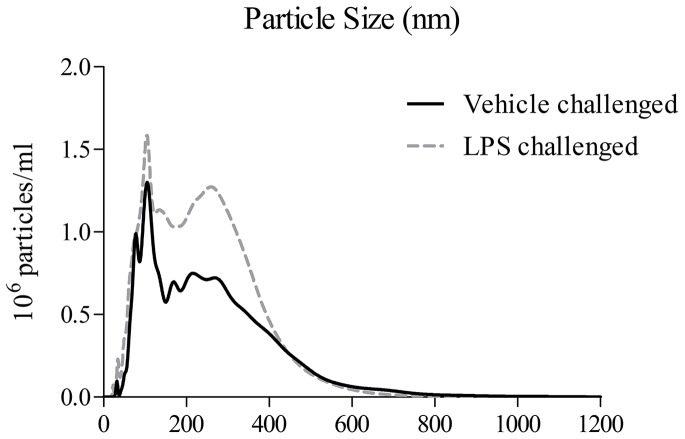
Determining if a bacterial mimetic (LPS) can cause the release of EVs in the lung – Nanosight imaging. Mice were challenged with the aerosolised vehicle of endotoxin-free saline or LPS (1 mg/ml) in Perspex chambers for 30 minutes. Animals were sacrificed and BALF obtained 6 hours after challenge. The samples were then centrifuged (900 g) to remove the white blood cells and debris. The presence of EVs was imaged using Nanosight technology.

When we prepared the EV fractions from mice challenged with the bacterial mimetic they appeared to contain more EV-like structures when compared to the saline challenged controls (Examples of the imagines are in Figure 3). Furthermore, assessing the cell-free BALF samples using Nanosight technology showed that the mice challenged with LPS had more particles around the size of EVs in their airways (20–1000 nM, Figure 4).

**Figure 5:**
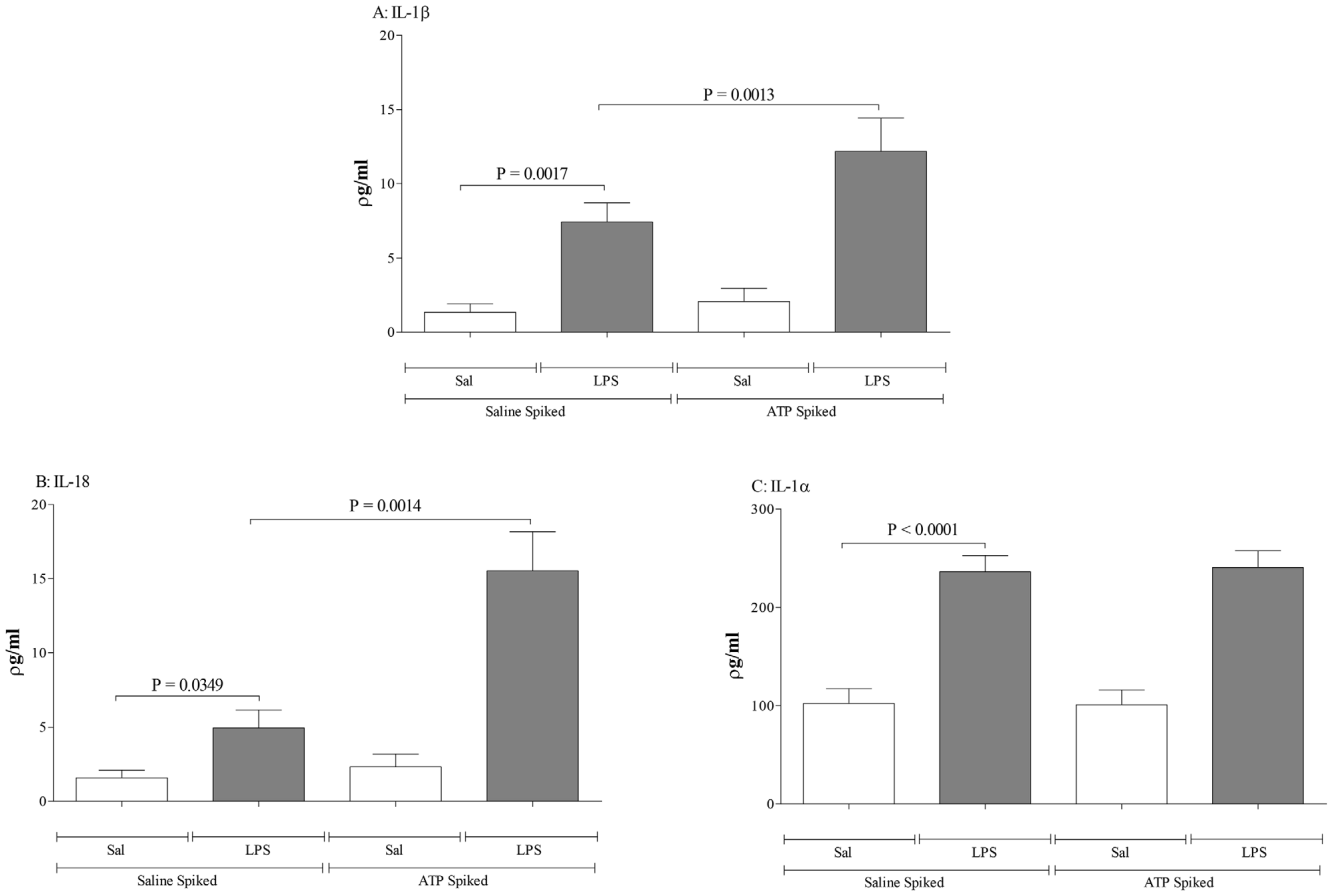
Determining if a bacterial mimetic (LPS) can cause the release of EVs in the lung – Cytokine release. Mice were challenged with the aerosolised vehicle of endotoxin-free saline or LPS (1 mg/ml) in Perspex chambers for 30 minutes. Animals were sacrificed and BALF obtained 6 hours after challenge. The samples were centrifuged (900 g) to remove the white blood cells and debris, and then treated with vehicle (PBS) or ATP*γS* (10^−3^ M), and incubated for a further 4 hours and subsequent cytokine release was analysed by ELISA. Data shown as mean +/− S.E.M. (A: IL-1β, B: IL-18, C: IL-1α).

**Figure 6:**
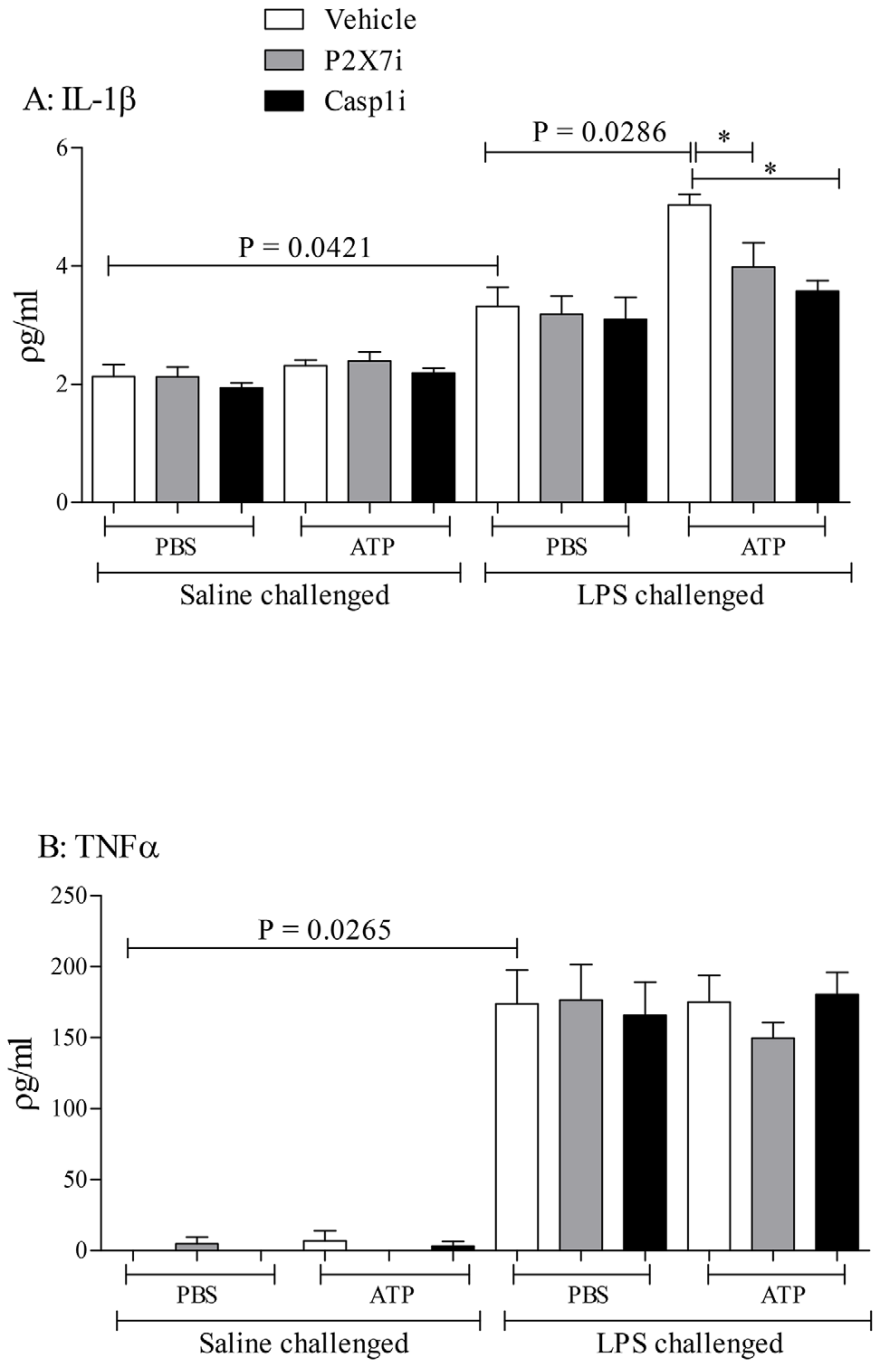
Determining if a bacterial mimetic (LPS) can cause the release of EVs in the lung – Signalling. Mice (n = 6 per group) were challenged with the aerosolised vehicle of endotoxin-free saline or LPS (1 mg/ml) in Perspex chambers for 30 minutes. Animals were sacrificed and BALF obtained 6 hours after challenge. The samples were then centrifuged (900 g) to remove the white blood cells and then pre-treated with inhibitors (P2X_7_ antagonist A 438079 (10^−6^ M) or caspase-1 inhibitor VX 765 (10^−7^ M)) and incubated for 1 hour. Samples were then treated with vehicle (PBS) or ATP*γS* (10^−3^ M), and incubated for a further 4 hours and subsequent cytokine release was analysed by ELISA. Data shown as mean +/− S.E.M. (A: IL-1β, B: TNFα). *  = P = 0.0138 (One way ANOVA followed by a Bonferroni's Multiple Comparison test).

LPS challenging the mice caused an increase in IL-1β, IL-18 and IL-1α in the BALF as expected (Figure 5). Stimulating the cell-free BALF with ATP resulted in a marked increase in IL-1β and IL-18, but not IL-1α (Figure 5). Pharmacological assessment of this increase showed that the ATP induced exacerbation was dependent on P2X_7_ and caspase 1 (Figure 6). The levels of TNFα, a cytokine not thought to be linked to the ATP-P2X_7_-Caspase 1 axis, were not altered as expected (Figure 6).

**Figure 7:**
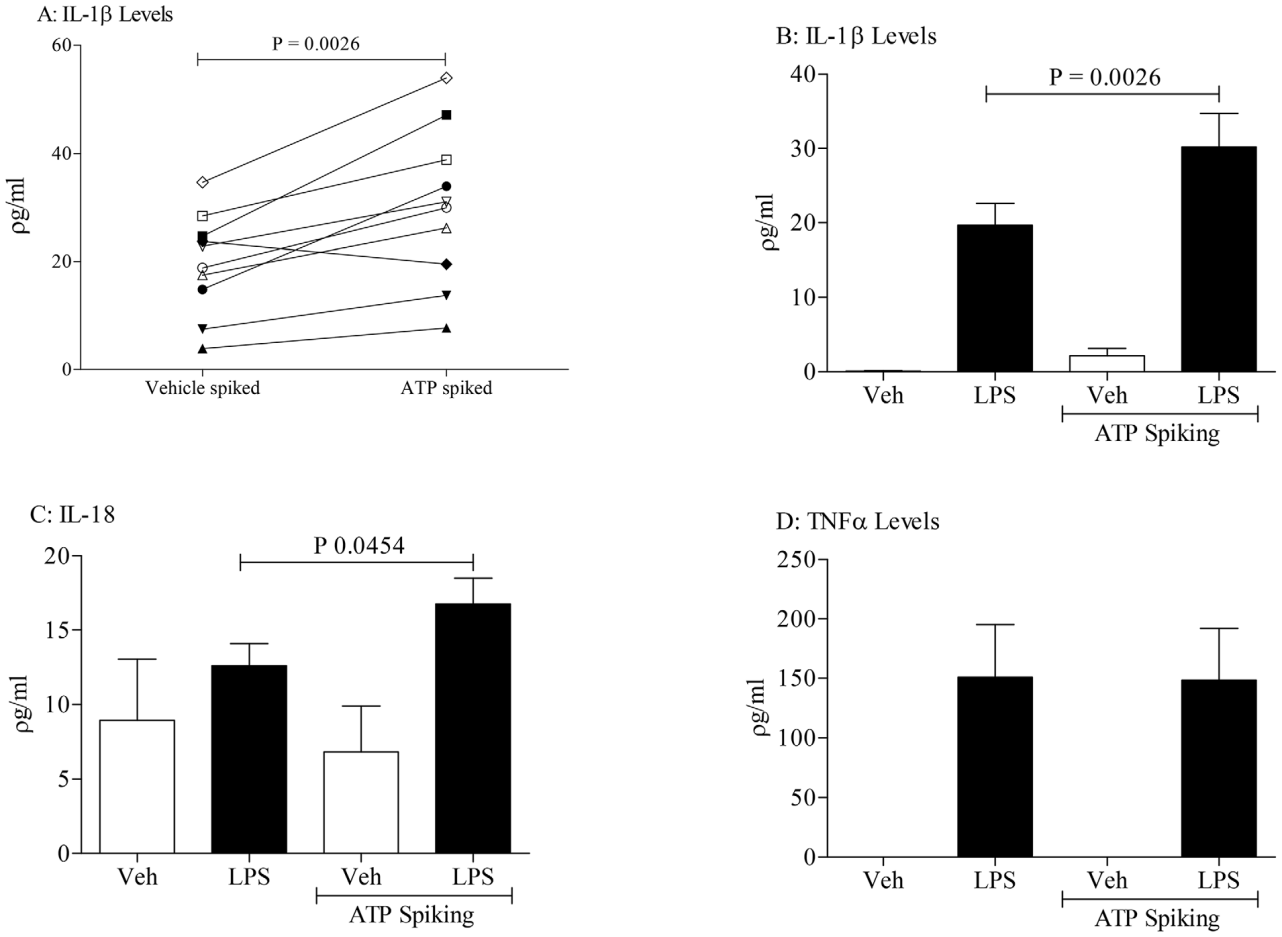
Human translation data: exogenous ATP increases IL-1β/IL-18 level in samples collected from LPS challenged healthy subjects. Healthy subjects were challenged with inhaled LPS and BALF was collected 6(10^−3^ M) and incubated for 4 hours; cytokine release was analysed by ELISA. Panel A shows the paired IL-1β data. Panel B, C and D represents the levels of IL-1β, IL-18 and TNFα, respectively. Data shown as mean +/− S.E.M. Statistical analysis using a paired T-test.

In an attempt to translate our pre-clinical findings, we collected BALF samples from healthy human subjects challenged with LPS, removed the white cells and debris and then spiked the BALF with vehicle or ATP. As can be seen from Figure 7, ATP significantly increased IL-1β, but not TNFα, in the samples from the subjects challenged with LPS. Similarly, we measured an increase in IL-18 but not IL-1α after ATP spike in the BALF from LPS challenged subjects (Figure 7 and data not shown). Like the murine model samples, we did not detect any change in ATP levels in the human BALF after LPS challenge (data not shown).

**Figure 8:**
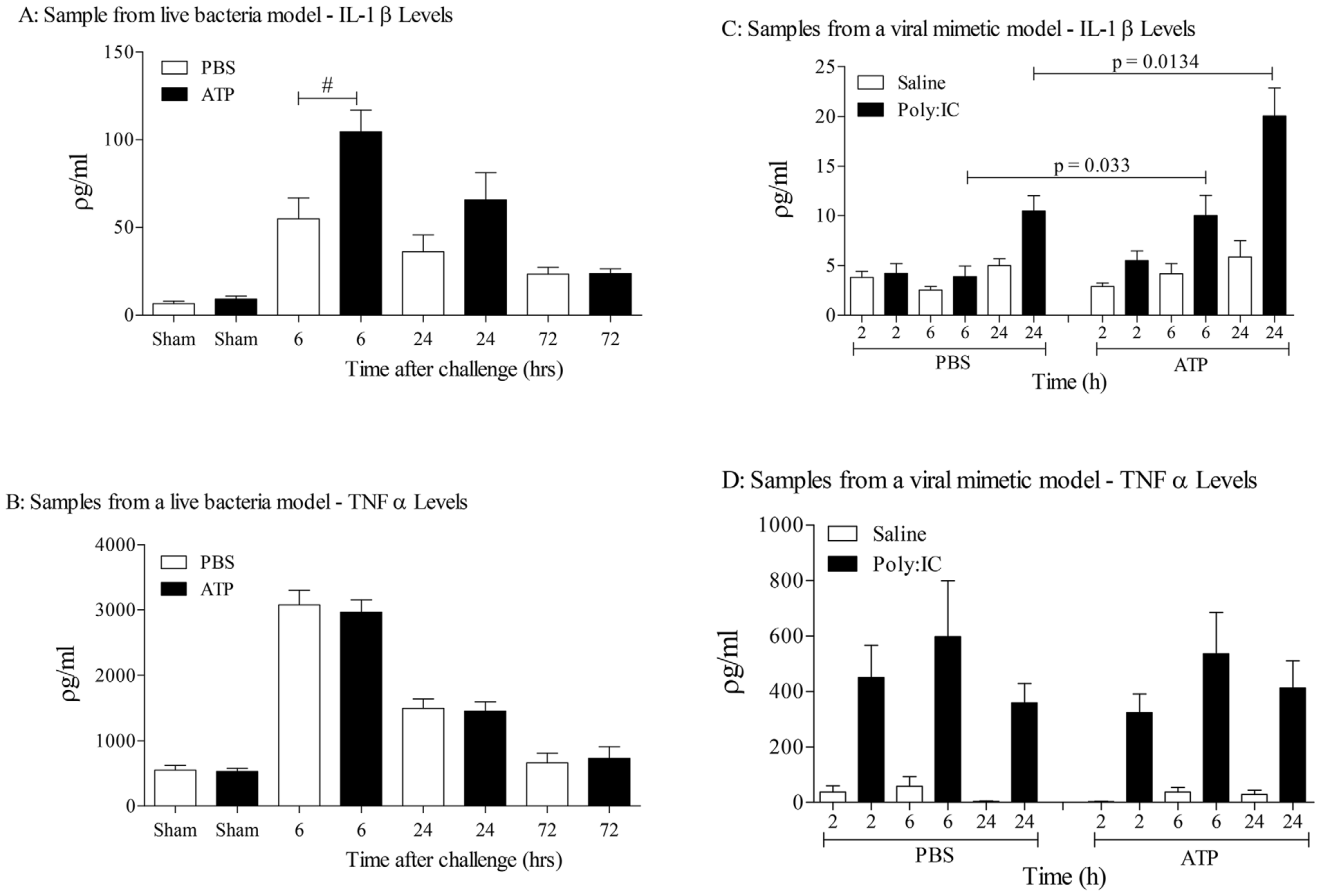
Determining if live bacteria or a viral mimetic can cause the release of EVs in the lung. Live bacterial model: Mice (n = 6 per group) were intranasally challenged with *Haemophilus influenzae* (1×10^7^ colony forming units serotype b) in sterile phosphate buffered saline (PBS). Mice were sacrificed and BALF samples were collected at 6, 24 and 72 hours. The samples were then centrifuged (900 g) to remove the white blood cells and then treated with vehicle (PBS) or ATP*γS* (10^−3^ M), incubated for 4 hours and analysed by ELISA. Data shown as mean +/− S.E.M. (A: IL-1β, B: TNFα). #  = P = 0.0206 (Mann-Whitney). Viral mimetic model: Mice (n = 6 per group) were challenged with vehicle (saline, approximately 25 µl per nostril) or the viral mimetic Poly I:C (0.6 mg/ml) under inhaled isoflurane. Mice were sacrificed and BALF samples were collected at 2, 6 and 24 hours. The samples were then centrifuged (900 g) to remove the white blood cells and then treated with vehicle (PBS) or ATP*γS* (10^−3^ M), incubated for 4 hours and analysed by ELISA. Data shown as mean +/− S.E.M. (C: IL-1β, D: TNFα).

To demonstrate that actual live bacteria could trigger EV release, we used BALF samples from a H. *influenza* bacterial challenge model. The inflammatory response observed in the model was typical of that reported previously [36]. As can be seen from Figure 8 (left panels) ATP spiking the samples increased the levels of IL-1β, and not TNFα, suggesting there appears to be functional EVs present in the airways of bacterially infected animals.

Similarly, in a model that uses a viral mimetic, poly I:C, which resulted in a typical inflammatory response [35], ATP spiking increased levels of IL-1β (Figure 8, right panels). Thus we are confident that bacterial and viral infections cause the release of EVs into the airway, and if there are high levels of ATP present in the airway (like in asthma and COPD), it could trigger the release of IL-1β and IL-18.

### Determining whether the ATP/P2X_7_ axis is central to the exacerbation response *in vivo*

**Figure 9:**
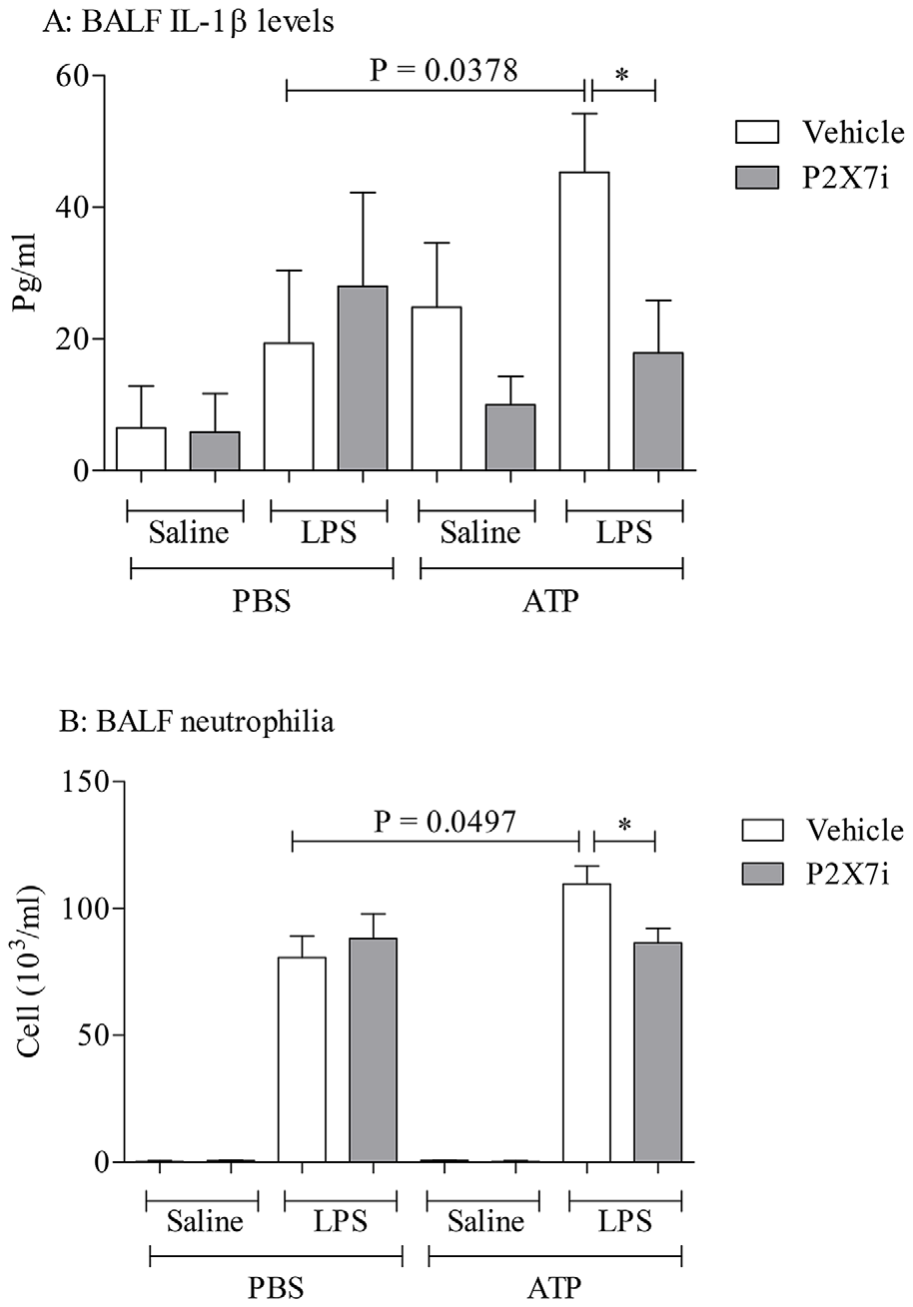
Determining whether the ATP/P2X_7_ axis is central to the exacerbation response *in vivo.* Mice (n = 8 per group) were challenged with the aerosolised vehicle of endotoxin-free saline or LPS (1 mg/ml) in Perspex chambers for 30 minutes. Four hours later the mice were intranasally dosed with saline (2 ml/kg) or ATPγs (0.001 mg/kg) whilst under light anaesthesia (4% isoflurane in oxygen). The mice received oral vehicle or P2X_7_ inhibitor, A438079, 30 minutes prior to the ATP challenge, 4 hours after the challenge and 1 hour prior to cull. Twenty four hours after the LPS exposure the mice were culled and lavaged. IL-1β (A) and neutrophil (B) numbers were measured in the BALF. Data shown as mean +/− S.E.M. An unpaired T-test was used for the statistical analysis. *  = P = 0.0378 (Panel A); *  = P = 0.0162 (Panel B).

Finally, an experiment was performed to determine if ATP could trigger P2X_7_ dependent exacerbation of LPS induced airway inflammation *in vivo*. Mice were challenged with LPS +/− ATP in the presence and absence of a P2X_7_ inhibitor. LPS challenge caused an increase in BALF IL-1β and neutrophilia; this was exacerbated in the mice that received ATP (Figure 9). Whilst the P2X_7_ inhibitor, A438079, had no effect on neutrophil numbers after just LPS challenge (as shown before [28], the ATP exacerbated response was attenuated by the inhibitor (Figure 9).

## Discussion

Exacerbations of both asthma and COPD can be fatal and represent a growing concern to patients and healthcare providers [6], [8]. These episodes are usually associated with viral and bacterial infections which enhance the existing inflammatory status, and in turn compromise lung function [9], [11]. We propose that these exacerbations could be caused by the increased levels of ATP in the lungs of these patients. Increased levels of ATP could activate the P2X_7_/caspase 1 axis in EVs resulting in the release of IL-1β/IL-18, and subsequent increase in inflammatory and disease status. To investigate this, we wanted to demonstrate that infective mimetics/agents could cause the release of EVs in the airway and that these EVs are functional/viable i.e. they can be triggered into releasing IL-1β and IL-18 in response to ATP.

It had been previously shown that exposure to smoke increases ATP levels in the BALF from mice [27] and that co exposure with smoke and the bacterial mimetic, LPS, led to an enhanced neutrophilic response which was similar in profile to that in exacerbating asthma and COPD sufferers [12], [13], [37], [38]. Together this suggested that the LPS exposure could be causing the release of EVs and the smoke-induced ATP could be causing the enhanced neutrophilia response via the release of enhanced levels of IL-1β/IL-18. To investigate this hypothesis we repeated the experiment using our murine model systems. The data clearly shows that combination of smoke and LPS leads to an exacerbated inflammatory response with IL-1β and neutrophilia levels enhanced. The ability to repeat the published data in a second species gave us confidence in the observation but more importantly indicated to us that mice are an appropriate species to use and enabled us to utilise our murine model systems. Furthermore, whereas we routinely observe an increase in ATP levels in our smoke model, as can be seen from Figure 1A we do not measure an increase in our LPS model. In corroboration, in these models we only observe an increase in caspase 1 activity (a downstream marker of ATP activity on the P2X_7_ receptor) in the smoke model [28]. The lack of LPS induced increase in ATP levels allows us to interpret the combination and ATP spiking data more readily.

Before further studying the possibility that respiratory infections can lead to the release of functional EVs in the airway, we first wanted to demonstrate the phenomenon in a cell based system. We found that the supernatant from cells treated with a bacterial mimetic had measurable levels of IL-1β, IL-18, MMP-9 and TNFα but only the levels of IL-1β and IL-18 were further increased in the presence of ATP. Furthermore, data showed that this enhanced release was P2X_7_-dependent. The role of EVs in this exacerbated response was confirmed when we compared supernatants that were ultracentrifuged, to remove the EVs (EV-deficient), with non-ultracentrifuged samples (EV-rich). Whereas the addition of ATP increased IL-1β and IL-18 levels in the EV–rich samples, we did not see a similar increase in the same samples when EVs were removed. These findings are similar to that observed by others which gave us confidence in our assessments and the use of IL-1β as a marker of the existence of functional/viable EVs [17], [20]–[23], [39], [40].

To begin to investigate whether respiratory infections caused the release of EVs we challenged mice with an inhaled bacterial mimetic. Assessment of the cell free BALF gave EM images that suggested an increase in particles after LPS challenge. This finding was quantified using Nanosight technology which gave a clear increase in particles in the samples harvested from LPS challenged mice. The size of these particles appeared to be in the 100–400 nM range, which is reminiscent of microvesicles, rather than exosomes [32], [33]. However, more detailed studies are required to establish the EV profile and, importantly, to determine the cellular source. While imaging data is useful it lacks the information required of these proof of concept studies i.e. that EVs are functional/viable, can release cytokines upon ATP stimulation via a P2X7-caspase 1 axis. To do this we used the bioassay as described. LPS challenge in mice caused an increase in the BALF IL-1α, IL-1β, IL-18 and TNFα levels as previously found [28]. The levels of IL-1β and IL-18, but not TNFα and IL-1α, were further increased when the cell-free BALF was spiked with exogenous ATP. This increase appeared to be dependent on both P2X_7_ and caspase 1 suggesting this axis was integral to the response. Whilst we do not know the exact mechanism that triggers the release of IL-1β and IL-18 (and not TNFα); the fact that it can be modulated by P2X_7_ and caspase 1 inhibitors suggests it is not simply through lysis. Together however, this data package does indicated that EVs are released and they are functional. Furthermore, using ATP induced IL-1β release was an appropriate surrogate marker for the biological fluid we were studying. By using this bioassay we were able to show that inhaled LPS can trigger the release of EVs in humans. This suggests that the data obtained in our murine model systems is translatable. Thus additional experiments were performed on other murine airway infection systems. We were able to show that as well as a bacterial mimetic a live bacteria challenge (H. influenzae, a gram-negative bacterium) also caused the release of EVs. Furthermore a viral mimetic, poly:IC, also triggered a similar response. This suggested to us that, as hypothesised, respiratory infections, whether it is bacterial or viral (both associated with exacerbation episodes in human patients), can trigger the release of EVs into the airway. And that if the levels of ATP are high, as described in patients with asthma and COPD, the EVs can release IL-1β and IL-18. Together this data set strongly suggests that infective agents in the airways cause the release of EVs and that ATP can drive them to release of IL-1β in a P2X_7_/caspase 1– dependent manner.

Finally, to determine whether the ATP/P2X_7_ axis is central to the exacerbation seen *in vivo*, we used a mechanistic model. The reason was that data in the disease model systems has shown that modulation of ATP levels or attenuation of P2X_7_ inhibits the inflammatory response [27], [28], [41], making interpretation of the exacerbation part of the model system difficult. Previously we have shown that the inflammation in the standard LPS model is not modulated by a P2X_7_ inhibitor [28], making it ideal to use as a mechanistic model for testing the importance of the P2X_7_ receptor in the ATP-driven exacerbation. The data generated clearly show that exogenous ATP caused a P2X_7_-dependent exacerbation of airway neutrophilia. The limitation of this *in vivo* assessment is that we can only infer from the *in vitro/ex vivo* data that the IL-1β released after ATP challenge is coming from EVs, there are other cellular sources in the airway like macrophages which we cannot rule out.

In our systems ATP alone did not cause airway neutrophilia; this is different to that reported by others [42]. Whilst we do believe that ATP can be a driving factor in airway inflammation, a good example is our published data in the smoke model [28], we think that ATP alone is not sufficient to cause cellular inflammation, other co-factors such as chemoattractants are required. Thus we are not surprised when the ATP alone did not cause neutrophilia, we cannot however explain the data published by Cicko *et al.* [42], it could perhaps be due to experimental design, health status of animals, etc. Another aspect of our findings that would appear to contradict published data is the lack of effect of the P2X_7_ receptor inhibitor on the LPS-only inflammation. Moncao-Ribeiro *et al.* published that P2X_7_ KO mice were protected from LPS challenge, with all aspects of the inflammatory response reduced in the GM mice compared to the wild type controls [43]. We have now repeated this study with litter matched controls and in agreement with our previous published findings [28], we find that in our model system the P2X_7_ receptor is not involved in the inflammation (data not shown). To us this result makes sense as it is what one would expect given there is no increase in ATP or the downstream marker, caspase 1 activity, in our model and it is well know that the LPS response triggers NF-kB driven inflammation. Again we cannot explain the difference in data but suggest it could perhaps be due to experimental design.

In summary, the results of this study indicate that respiratory infections, bacterial and viral, can trigger the release of functional EVs in mice and man. Furthermore, upon ATP activation, the EVs released IL-1β and IL-18 in a P2X_7_/caspase-1 axis dependent manner resulting in exacerbated neutrophilia. We suggest EVs and this signalling pathway could prove to be a viable therapeutic target in the future to improve the clinical outcomes of the exacerbations of asthma and COPD.
